# Over-Represented Senescent Keratinocytes in Hyperpigmented Spots Promote Melanocyte Activation via IGFBP3 and NGF

**DOI:** 10.3390/ijms262110724

**Published:** 2025-11-04

**Authors:** Tomohiro Hakozaki, Holly Rovito, Bradley B. Jarrold, John Snowball, Jiazhen Wang, Wenzhu Zhao, Timothy Laughlin

**Affiliations:** The Procter & Gamble Company, Mason Business Center, Mason, OH 45040, USA; rovito.ha@pg.com (H.R.); jarrold.bb@pg.com (B.B.J.); snowball.jm@pg.com (J.S.); wang.j.55@pg.com (J.W.); zhao.wh@pg.com (W.Z.); laughlin.lt@pg.com (T.L.)

**Keywords:** senescence, keratinocytes, melanocyte dendricity, melanin, hyperpigmented spots, cytokines, IGFBP3, NGF, p16

## Abstract

The occurrence and impact of cellular senescence on skin aging and hyperpigmentation is an ongoing area of exploration, encompassing both intrinsic and extrinsic stressors. Traditionally, research has focused on melanocyte and fibroblast senescence due to their slower turnover compared to keratinocytes. In this study, we identified the accumulation of p16, a senescence marker, in keratinocytes from biopsies of multiple spot types. We explored their impact using doxorubicin-induced senescent keratinocytes in vitro. Conditioned media from these senescent keratinocytes stimulated melanocyte dendricity, a hallmark of hyperpigmented spots. Transcriptomic analysis of senescent keratinocytes identified two key senescence-induced factors: Insulin-like Growth Factor-Binding Protein 3 (*IGFBP3*) and Nerve Growth Factor (*NGF*). IGFBP3 and NGF ligand treatment enhanced melanin synthesis by 33% and 17%, and dendricity by 23% and 14%, respectively, in human melanocyte cultures. These findings suggest that keratinocyte senescence contributes to spot formation by mediating melanocyte activation through IGFBP3 and NGF. Furthermore, we evaluated skincare ingredients such as sucrose dilaurate, glabridin, and niacinamide in neutral and low pH solutions, demonstrating their efficacy in reducing the secretion of these ligands, thereby offering potential cosmetic benefits. This study provides insights into the mechanisms of spot formation and highlights promising strategies for managing pigmentation disorders.

## 1. Introduction

Facial spots, such as solar lentigo, melasma, and post-inflammatory hyperpigmentation (PIH), are prevalent dermatological and cosmetic concerns. These hyperpigmented lesions arise from the overproduction and uneven distribution of melanin, leading to significant aesthetic and psychological impacts [1,2]. A critical factor besides melanin synthesis in these pigmentation patterns is melanocyte dendricity, which indicates the activation of melanocyte and facilitates melanosome transfer to keratinocytes, thereby contributing to the visible appearance of these spots [3,4]. Recent studies have confirmed enhanced dendricity in hyperpigmented spot areas, which promotes melanosome transfer and results in persistent hyperpigmentation [5,6,7,8]. Moreover, the microenvironment of spots, influenced by factors such as ultraviolet (UV) light exposure and hormonal changes, has been found to contribute to the increased melanocyte dendricity [9,10].

Cellular senescence is a state of irreversible growth arrest that occurs due to aging or in response to various stressors, such as DNA damage and oxidative stress. Senescent cells secrete a range of bioactive molecules, collectively known as the senescence-associated secretory phenotype (SASP), which can profoundly influence the tissue microenvironment [11,12,13,14]. In the skin, senescent fibroblasts have been shown to alter the extracellular matrix and modulate inflammatory responses, which can contribute to visible signs of aging, such as wrinkle formation and loss of elasticity [15,16,17,18]. Senescent fibroblasts have been implicated in increased collagen degradation and decreased synthesis, further exacerbating skin aging [19,20]. Additionally, senescent melanocytes have been implicated in dysregulated pigmentation and the development of age-related skin lesions [21].

While the impact of senescent cells on general skin aging is well-documented, their specific role in pigmentation, particularly in relation to facial spots, remains underexplored. Here, we present our findings on the accumulation of senescent cells, particularly keratinocytes, in the epidermis across multiple types of spots. By leveraging a senescent cell culture model, we discovered that senescent keratinocytes secrete insulin-like growth factor-binding protein 3 (IGFBP3) and nerve growth factor (NGF), which act as key paracrine factors for melanocyte activation, suggesting their direct role in enhancing pigmentation in spot areas. Additionally, we evaluated several skincare ingredients for their effectiveness in suppressing the release of these ligands, which would lead to a reduction in ligand-induced dendricity and melanin synthesis in melanocytes.

## 2. Results

### 2.1. Accumulated p16 in Multiple Spots

**Figure 1:**
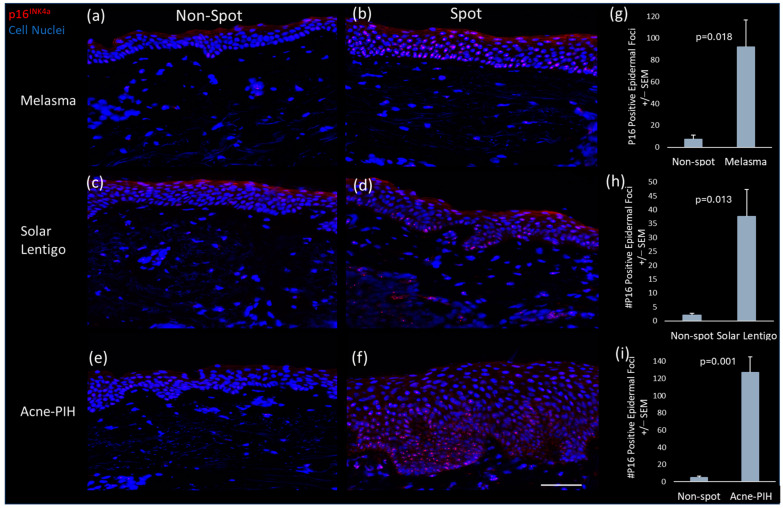
Senescence marker staining for paired non-spot and melasma, solar lentigo and acne-induced PIH biopsies. Representative images of P16^INK4a^ immunofluorescence staining (red) of non-spot (**a**,**c**,**e**) and spot (**b**,**d**,**f**) tissue. Image analysis quantification of total epidermal P16^INK4a^ foci in non-spot compared to (**g**) melasma, (**h**) solar lentigo and (**i**) acne-induced PIH spot biopsies. Minimal non-specific stratum corneum staining (red) was observed in multiple biopsies. Scale bar = 100 µm. Bars in the graphs indicate mean + SEM (N = 3 donors per spot type; *p*-value obtained from Student’s *t* test).

Immunofluorescent staining of p16^INK4a^, a recognized marker of cellular senescence [22,23,24], was performed on biopsy samples from spot and non-spot skin of Chinese females. The results revealed an accumulation of p16 ^INK4a^-positive cells in the epidermis of spot skin (Figure 1b,d,f) compared to non-spot skin (Figure 1a,c,e) from the same donors. The pattern was consistent across all three types of spots evaluated: melasma, solar lentigo, and acne-induced post-inflammatory pigmentation (acne-PIH). Quantitative image analysis confirmed a significantly higher number of p16^INK4a^-positive epidermal cells in spot areas compared to non-spot areas (Figure 1g,h,i), indicating an increased presence of senescent cells, predominantly keratinocytes, in the epidermis.

### 2.2. Proof of Senescent Phenotype of Doxorubicin-Treated Keratinocytes

**Figure 2:**
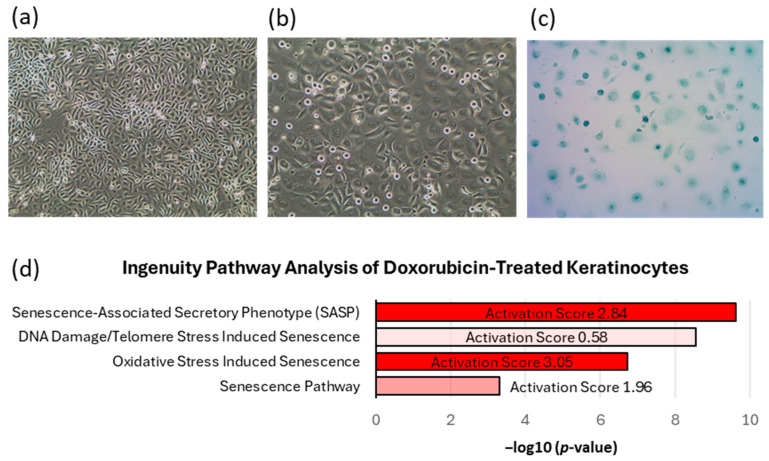
Validation of senescent profile of doxorubicin-treated HaCaT keratinocytes. HaCaT keratinocytes were treated with 500 nM doxorubicin for 24 h, followed by removal, washing, replacement with fresh media, and harvesting 48 h later. Phase contrast microscopic images (10×) show (**a**) untreated HaCaT keratinocytes and (**b**) doxorubicin-treated HaCaT keratinocytes, revealing senescent morphology characterized by an abnormal and flattened shape at 72 h. (**c**) β-galactosidase staining (blue) of doxorubicin-treated HaCaT keratinocytes (10×), confirming senescence induction. (**d**) RNAseq analysis was performed 24 h after doxorubicin treatment, comparing treated and untreated HaCaT keratinocytes (N = 4). Senescent related pathways were significantly upregulated (*p* < 0.001) based on differentially expressed genes, using Ingenuity Pathway Analysis. Activation scores correspond to the direction and intensity a pathway is predicted to be altered and is based on gene expression changes. Corresponding *p*-values (x-axis) are based on the number of genes in each pathway altered. Differentially regulated genes: DEseq2 Negative Binomial Q-value <0.01, |FC| > 2, and FPKM > 1 in over half of the biological replicates in one condition being compared.

To investigate the role of senescent epidermal keratinocytes in skin pigmentation, we established senescent HaCaT keratinocytes by treating them with doxorubicin through modification of a previously described protocol [25,26]. Microscopic observation of doxorubicin-treated keratinocytes confirmed a senescent morphology characterized by an abnormal and flattened shape [15] compared to untreated HaCaT keratinocytes (Figure 2a,b). Along with morphology changes, positive β-galactosidase staining confirmed senescence induction of doxorubicin-treated keratinocytes (Figure 2c). Additionally, RNA sequencing analysis confirmed transcriptional activation of genes associated with known senescence related pathways and interferon signaling 24 h after doxorubicin treatment (Figure 2d and Figure S1).

### 2.3. Senescent Keratinocyte-Conditioned Media Enhance Melanocyte Dendricity

**Figure 3:**
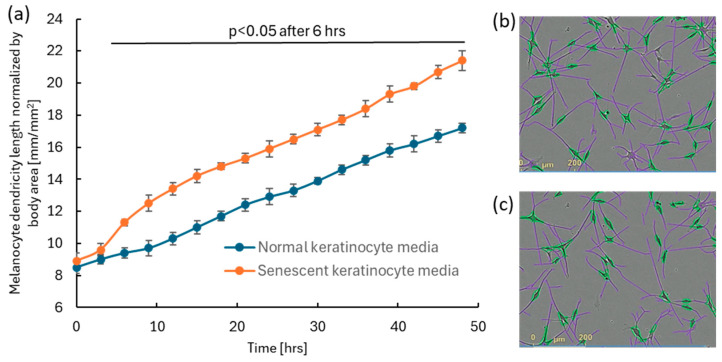
The addition of senescent keratinocyte-conditioned media to human melanocyte cultures enhanced dendricity formation compared to cultures treated with normal keratinocyte-conditioned media. Senescent keratinocyte-conditioned media were induced by 500 nM doxorubicin. 25% of either senescent or control media was added to the human melanocyte culture. (**a**) Significant enhancement in dendricity (N = 24; *p* < 0.05; a mixed effect model followed by Bonferroni’s multiple comparison test) was observed 6 h after media treatment. Error bar indicates SD. Representative images of detected melanocytes and their dendricity analyzed using the IncuCyte ZOOM NeuroTrack software (purple: dendrite, green: melanocyte cell body) for (**b**) induced melanocytes and (**c**) normal melanocytes at 48 h.

Media collected from doxorubicin-induced senescent keratinocytes, as well as from normal keratinocytes, were used to treat monolayer cultures of human primary melanocytes. An increase in melanocyte dendricity was observed in cultures treated with senescent keratinocyte-conditioned media compared to those treated with normal keratinocyte media (Figure 3). The result suggests that senescent keratinocytes secrete factors that stimulate the formation of melanocyte dendricity, a hallmark feature of facial spot areas [5].

### 2.4. Identification of Potential Ligands to Stimulate Melanocyte Dendricity via Transcriptome Analysis of Senescent Keratinocytes

**Figure 4:**
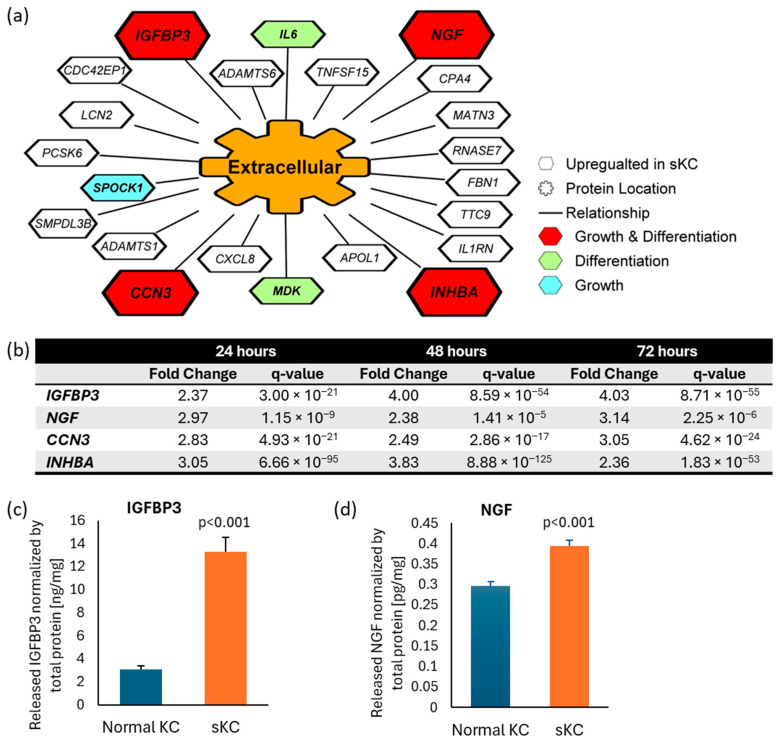
Identification of IGFBP3 and NGF as plausible ligands released from senescent keratinocytes to enhance melanocyte dendricity. (**a**) RNAseq analyses of doxorubicin-induced senescent keratinocytes compared to non-treated normal keratinocytes was performed to identify potential ligands linked to dendricity. Twenty-two genes annotated as “extracellular” were selected, all of which are upregulated at all three timepoints compared to time-matched untreated controls, using the criteria of |FC| > 2, q-value < 0.01 (N = 4). Four genes associated with “cell growth” and “positive regulation of cell differentiation” were prioritized (in red). (**b**) Among these four genes, *IGFBP3* and *NGF* showed the highest fold change induction at 72 h thus were selected for further analyses. Quantification of (**c**) IGFBP3 and (**d**) NGF protein released in the media by ELISA from senescent keratinocytes and normal keratinocytes. Bar indicates mean + SD (N = 6; *p*-value obtained from Student’s *t* test). KC: keratinocyte; sKC: senescent keratinocyte.

To identify potential ligands which stimulated the observed melanocyte dendricity in senescent keratinocyte-conditioned media, we performed a transcriptome analysis of doxorubicin-induced senescent keratinocytes compared to the time matched control keratinocytes without doxorubicin treatment 24, 48 and 72 h after treatment. 22 genes that code for “extracellular”-located proteins or secreted proteins were upregulated at all three timepoints (Figure 4a). Of those genes, four (*IGFBP3*, *INHBA*, *NGF*, *CCN3*) are associated with the biological processes of cell differentiation (Gene Ontology: 0045597) and cell growth (Gene Ontology: 0016049), suggesting a higher potential to affect melanocyte morphology, including dendricity. Among these four genes, we selected *NGF* and *IGFBP3* for further analysis, as they were the top two upregulated genes at 72 h timepoint based on fold change (Figure 4b). To confirm the presence and release into the media, we analyzed IGFBP3 and NGF protein by using Enzyme-Linked Immunosorbent Assay (ELISA). The result confirmed enhanced release of IGFBP3 and NGF proteins into media from senescent keratinocytes compared to normal keratinocytes (Figure 4c,d).

### 2.5. IGFBP3 and NGF Stimulate Melanocyte Dendricity and Melanin Production

**Figure 5:**
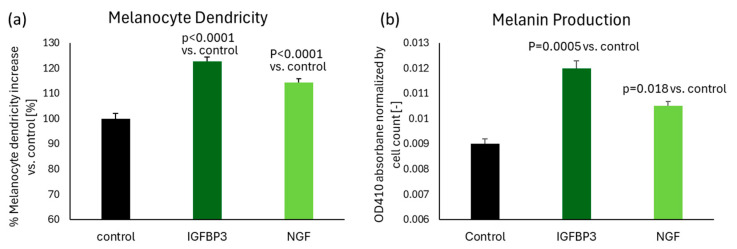
The addition of IGFBP3 or NGF significantly stimulated melanin production by 33% and 17%, and dendricity formation by 23% and 14%, respectively, in human melanocytes. (**a**) Melanocyte dendricity length was measured after 48 h of treatment with either 1 ng/mL IGFBP3 or NGF, compared to untreated control. N = 16 for each group. (**b**) Melanin production was quantified after 72 h of treatment with either 1 ng/mL IGFBP3 or NGF, compared to untreated control. N = 4 for each group. Bar indicates mean + SD (*p*-value obtained from one-way ANOVA followed by Tukey’s multiple comparison test).

To determine whether IGFBP3 and NGF serve as key ligands that induce melanocyte dendricity, each ligand protein was added separately to human melanocyte cultures. Results demonstrated that both IGFBP3 and NGF significantly stimulate dendricity formation in melanocytes (Figure 5a). Additionally, we assessed the ability of these ligands to enhance melanin production in human melanocytes. The results revealed that both ligands significantly increased melanin production as well (Figure 5b), suggesting these ligands, released from senescent keratinocytes, contribute to the activation of melanocytes dendricity and melanin production in spots.

### 2.6. Effect of Skin Care Ingredients on Suppressing IGFBP3 and NGF Release from Senescent Keratinocytes

**Figure 6:**
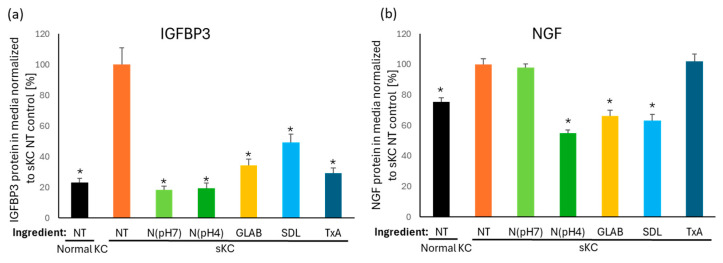
Skin care ingredients significantly suppressed the releases of IGFBP3 and NGF from doxorubicin-induced senescent keratinocytes. (**a**) IGFBP3 and (**b**) NGF protein levels in media from senescent keratinocytes were measured following 72 h of treatment with various skin care ingredients: N(pH7): 1/50 dilution of 5% niacinamide at pH 7; N(pH4): 1/50 dilution of 5% niacinamide at pH 4 adjusted with hydrochloric acid; GLAB: 0.00125% Glabridin, SDL: 0.001% sucrose dilaurate; TxA: 0.1% tranexamic acid. For each ingredient, the maximum concentration without affecting cell viability was tested. The detected amounts were normalized to sKC NT control levels. Bar indicates mean + SD (N = 6; * *p* < 0.05 vs. sKC NT control, one-way ANOVA followed by Tukey’s multiple comparison test). KC: keratinocyte; sKC: senescent keratinocyte; NT: no treatment.

To mitigate the release of IGFBP3 and NGF from senescent keratinocytes, we screened various skin care ingredients and identified that niacinamide, glabridin, sucrose dilaurate and tranexamic acid effectively suppressed the release of one or both ligands. All four ingredients strongly suppressed IGFBP3 release to levels similar to the innate levels released by normal keratinocytes (Figure 6a). Glabridin and sucrose dilaurate also effectively suppressed NGF release, whereas tranexamic acid did not. Interestingly, niacinamide did not suppress NGF release, when prepared in a neutral pH solution at 5% and added at a 1/50 dilution (= 0.1% niacinamide); however, when niacinamide was prepared in a pH 4 solution (ranging 3.8–4.4, adjusted with hydrochloric acid) and added at same dilution, it strongly suppressed NGF release.

## 3. Discussion

In the past few decades, the mechanism of skin cellular senescence and their impact on skin aging have been extensively researched and well documented [27,28]. However, the specific role of senescent cells in skin pigmentation has been explored in relatively few studies, primary in vitro. A few papers have reported that senescent fibroblasts influence pigmentation by affecting melanocyte activity. For instance, Duval et al. [29] demonstrated that photo-aged dermal fibroblasts exhibiting a senescent phenotype increased melanin production more than young, non-photo-exposed fibroblasts in a pigmented reconstructed skin model, suggesting that senescent fibroblasts can alter melanocyte behavior and melanin production. Similarly, Kim et al. [30] reported that senescent fibroblast-derived growth differentiation factor 15 (GDF15) induces skin pigmentation in a co-culture model of melanocytes and senescent fibroblasts, highlighting a paracrine signaling mechanism that enhances melanocyte activity. In addition to fibroblasts, senescent melanocytes themselves exhibit changes that impact pigmentation. Park et al. [31] identified glycolytic metabolism alterations in senescent melanocytes that cause melanosome transport dysfunction, speculated to be due to impaired mitochondrial function and metabolic reprogramming. Furthermore, the role of senescent keratinocytes in pigmentation was recently reported [24] demonstrating that senescent keratinocytes accumulate melanin due to impaired energy metabolism, suggesting a direct contribution to persistent pigmentation. These findings collectively underscore the multifaceted role of senescent cells in regulating skin pigmentation through various cellular and molecular mechanisms.

Building on the understanding of senescent cells in pigmentation, two recent studies have indicated their localization in the dermis of hyperpigmented spots, suggesting a potential link between cellular senescence and spot formation. Yoon et al. [32] demonstrated that solar lentigo exhibits an increasing proportion of senescent fibroblasts, with the loss of stromal cell-derived factor 1 (SDF1) potentially contributing to melanogenesis by altering the local microenvironment. Similarly, Wan et al. [33] proposed that senescent fibroblasts might incite melanocytes to enter a hyperactive state, exacerbating the pigmentation observed in melasma. Although these studies reported the presence of senescent cells in the dermis of spots and their potential impact on melanocyte activities, they did not report the existence of senescent cells in the epidermis of spots. We have recently reported that, in multiple types of facial spots—including solar lentigo, melasma, PIH, pigmented seborrheic keratosis, and freckles—*CDKN2A* gene expression is elevated in the collected spot biopsies compared to adjacent healthy skin, with this increase occurring particularly within the epidermis rather than the dermis [5]. In the present study, we demonstrated that the p16^INK4a^ protein, encoded by the *CDKN2A* gene, is significantly accumulated in three types of hyperpigmented spots—solar lentigo, melasma, and acne-induced PIH—as evidenced by immunofluorescent staining, with its prevalence predominantly in the epidermis as shown in Figure 1. These results emphasize the role of senescent epidermal cells in diverse spot types, highlighting a common biological theme.

Enhanced melanocyte dendricity is reported as a common hallmark of multiple types of hyperpigmented spots [5]. To explore the relationship between melanocyte dendricity and senescent keratinocytes, we conducted a series of experiments. For our studies, we leveraged doxorubicin-induced senescent keratinocytes. Doxorubicin induction is well-documented senescent model and widely used for the research of aging and skin diseases, as senescent cells accumulate in aged tissues and contribute to chronic inflammation and tissue dysfunction [34,35]. For proof of concept, we examined the effects of conditioned media from doxorubicin-induced senescent keratinocytes on melanocytes and observed a marked stimulation of dendricity. This finding suggests that senescent keratinocytes secrete factors that activate melanocyte dendricity. To identify these factors, we conducted RNA sequencing on senescent keratinocytes and identified potentially externally releasable 22 candidate genes with upregulated expression. Of these, four are associated with the biological processes of both cell differentiation and cell growth, indicating they have a biological basis for affecting cell morphology including dendricity. Among these four, we focused on the two most strongly expressed genes at 72 h, *IGFBP3* and *NGF*. Later, we confirmed their novel presence in the senescent keratinocyte media at the protein level. These ligands are known to play roles in cellular communication and growth, and their presence in the media underscores their potential contribution to the observed enhancement of melanocyte dendricity.

IGFBP3 and NGF are pivotal proteins with distinct roles in cellular signaling and function. IGFBP3 is a key component of the insulin-like growth factor (IGF) pathway, known for binding various partners such as IGFs, epidermal growth factor receptor, and transforming growth factor beta receptors [36]. Interestingly, IGFBP3 has also been reported to interact with unknown membrane proteins [36], suggesting a broader spectrum of cellular effects. Recent study has unveiled that senescent keratinocytes release IGFBP3 in organotypic 3D skin models [37], providing insights into its role in epidermal aging. However, prior to our study, there was no evidence indicating IGFBP3’s impact on human melanocyte activity. Our findings are the first to demonstrate that IGFBP3, secreted from senescent keratinocytes, significantly enhances melanocyte dendricity and melanin synthesis.

NGF is a vital protein that promotes the growth, survival, and function of nerve cells, and is known to induce neurite outgrowth and dendricity in nerve cells [38]. Previous research has shown that keratinocyte-derived NGF can induce dendricity in melanocytes as well [39,40]. Our study confirms that NGF, which was over-secreted from senescent keratinocytes, can enhance melanocyte dendricity and melanin production. These findings expand the known roles of IGFBP3 and NGF, highlighting their contributions to the cellular dynamics within hyperpigmented spots and presenting new avenues for understanding and targeting pigmentation disorders.

Lastly, we explored whether specific skincare ingredients known to provide visible skin lightening benefits could mitigate the release of IGFBP3 and NGF from senescent keratinocytes. Our findings revealed that niacinamide [41], glabridin [42], sucrose dilaurate [9], and tranexamic acid [43,44], all reported to reduce hyperpigmented spots, effectively suppressed the secretion of either or both of these ligands, thereby reducing their stimulatory effects on melanocyte dendricity and melanin production in vitro. Therefore, our findings provide another potential mechanism of action for these skin lightening ingredients. Notably, niacinamide exhibited pH-dependent activity, with more pronounced effects in acidic conditions. Indeed, niacinamide at pH 4 demonstrated the strongest suppression of NGF and IGFBP3 among the tested ingredients. Interestingly, only niacinamide at pH 4 showed suppression of NGF, while niacinamide at neutral pH did not, highlighting the importance of formulation conditions in enhancing ingredient efficacy.

Despite some limitations, this study generates future directions for understanding the role of senescent cells in hyperpigmented spots. First, the evidence for the overexpression and causal role of IGFBP3 or NGF in vivo skin remains limited. Given the anticipated low abundance and potential localization of these ligands, single-cell and spatial analyses of spot biopsies would be well-suited to confirm our findings and are considered in future work. Second, while we identified 22 plausible candidate genes released from senescent keratinocytes, we focused on two novel ligands, IGFBP3 and NGF, for in-depth investigation. The other identified genes, particularly CCN3 and INHBA, merit further exploration to understand their roles in melanocyte activity. Third, our observation of the accumulation of senescent cells in the epidermis of spot skin led us to predominantly discuss the role of senescent keratinocyte derived factors. However, as referenced in previous studies, factors derived from senescent fibroblasts in the dermis may also be involved [30,32]. To more accurately replicate the physiological conditions of in vivo skin, further research should focus on establishing integrated 2D or 3D models that replicate the interplay between senescent cells and their derived factors, such as senescent keratinocytes and fibroblasts, which may act additively or synergistically. Lastly, although we demonstrated the suppression of IGFBP3 and NGF by these skin lightening ingredients, the molecular mechanisms underlying this action and clinical translations to reduce hyperpigmented spots remain unknown and warrant further investigation.

In conclusion, our study revealed the accumulation of senescent keratinocytes in three types of hyperpigmented spots—solar lentigo, melasma, and acne-induced PIH. The study also elucidated the potential role of senescent keratinocytes, highlighting the critical involvement of IGFBP3 and NGF in enhancing melanocyte dendricity and melanin production, which are key features common to multiple types of hyperpigmented spots. This novel mechanism sheds light on the impact of accumulated senescent keratinocytes in spot formation, suggesting a potential common biological theme across these conditions. Furthermore, we demonstrated that several skincare ingredients, which are reported to reduce spots, can suppress the secretion of these ligands, indicating an additive possible mechanism of action of these ingredients to mitigate the appearance of spots. Overall, this study provides valuable insights into the mechanisms underlying spot formation and highlights a promising approach for improving the appearance of facial spots.

## 4. Materials and Methods

### 4.1. Chemicals, Reagents, and Cell Lines

Cell culture media and supplements were purchased from Thermo Fisher Scientific (Waltham, MA, USA), including EpiLife with 60 μM calcium (Cat. No. MEPI500CA), gentamicin/amphotericin B (500X; Cat. No. 50-0640), HKGS (100X; Cat. No. S-001-5), trypsin/EDTA solution (TE; Cat. No. R001100), trypsin neutralizer solution (TN; Cat. No. R002100), penicillin/streptomycin (10,000 U/mL, 100X; Cat. No. 15140122), and DPBS (Cat. No. 14190250). ViaStain AO/PI staining solution was purchased from Nexcelom Bioscience (Cat. No.: CS2-0106-5 mL, Lawrence, MA, USA). AccuGene 1× PBS was purchased from Lonza (Cat. No. 51225, Alpharetta, GA, USA). Doxorubicin hydrochloride was purchased from Tocris (Cat. No. 2252/10, Minneapolis, MN, USA), niacinamide was purchased from Sigma-Aldrich (Cat. No. N5535, St. Louis, MO, USA), glabridin was purchased from Jiangsu Tiansheng Pharmaceutical Co. (Jurong, China), tranexamic acid was purchased from Caymen Chemical Co. (Cat. No. 19193, Ann Arbor, MI, USA), and sucrose dilaurate (mixture of sucrose laurate and dilaurate) was purchased from BASF (Ludwigshafen, Germany). IGFBP3 and NGF proteins were purchased from R&D Systems (Minneapolis, MN, USA: Cat# 675-B3-025 and 256-GF). Other chemicals used were purchased from Sigma-Aldrich (St. Louis, MO, USA), including DMSO (Cat. No. D8418-100 mL).

### 4.2. Subject Population and Biopsy Collection for Facial Spots

2 mm biopsies of three types of facial spots (solar lentigo, post-inflammatory hyperpigmentation (PIH) induced by acne, light symptom melasma; diagnosed by the study dermatologist) as well as adjacent non-spot tissue were collected from Chinese women aged 20–70 years using protocols approved by the RCRC Independent Review Board at Biometrix in San Francisco, California. All subjects signed written informed consent before undergoing study procedures. Collected biopsies were embedded in Optimum Temperature Compound (Sakura Finetek, Torrance, CA, USA) before freezing over liquid nitrogen and stored at −80 °C until cryostat sectioning at 7 µm.

### 4.3. CDKN2A/p16^INK4a^ Immunohistology for Senescence Assessment

Histological assessment of senescence was performed on a total of 9 subjects, comprising 3 pairs of representative spot and non-spot skin samples for each of 3 spot types (melasma, solar lentigo, and acne-PIH). 7 µm fresh frozen sections of each subject’s paired spot and non-spot tissue were processed in parallel through fixation in ice cold acetone for 10 min at −20 °C, washed in phosphate-buffered saline (PBS) and incubated for 1 h at room temperature (RT) in 10% normal goat serum in PBS (Cell Signaling Tech, Danvers, MA 5425S). Sections were incubated for 1 h at RT with an anti-CDKN2A/p16^INK4a^ antibody (Abcam, Waltham, MA, USA, ab108349 1:500), washed in PBS, incubated with Alexa Fluor 555 conjugated goat anti-rabbit antibodies (Abcam, Waltham, MA, ab150086 1:1000) for 30 min at RT. After washing in PBS, sections were counterstained with 4′,6-diamidino-2-phenylindole (DAPI) using NucBlue Fixed Cell Stain Ready Probes Reagent (Invitrogen, Carlsbad, CA, USA). Sections stained without the primary antibody served as negative controls and displayed no non-specific staining. For comparison, fluorescent images of non-spot and spot pairs were captured with a Zeiss Observer.Z1 microscope (Carl Zeiss Microimaging, Jena, Germany) at equal gamma values, pixel range and exposures. Two separate fields of view were captured for each biopsy, and the epidermal p16^INK4a^ positive staining foci were segmented and counted using Image Pro-Software (version 10, Media Cybernetics, Rockville, MD, USA). Statistical significance (N = 3 for each spot type) was established utilizing mean values of spot biopsies compared to their non-spot controls using Student’s *t*-test, with a significance threshold set at *p* ≤ 0.05.

### 4.4. Keratinocyte Cell Culture General

Human HaCaT Keratinocytes were purchased from AddexBio (San Diego, CA, USA). The cells were cultured in Epilife medium with 60 uM calcium (Thermo Fisher) and supplemented with Human Keratinocyte Growth Supplement (Thermo Fisher) and gentamicin/amphotericin B 500X solution (Thermo Fisher) in T-75 flasks (Corning). The cultured cells were maintained at 37 °C, 5% CO_2_, and 90% relative humidity.

### 4.5. Preparation of Doxorubicin-Induced Senescent Keratinocytes

HaCaT keratinocytes were seeded into 12 well tissue culture plates at a density of 2 × 10^5^ cells per well and maintained in Epilife medium at 37 °C, 5% CO_2_, and 90% relative humidity for four days, with medium changes every two days. On the fourth day, senescence was induced with 500 nM of doxorubicin hydrochloride. After 24 h, doxorubicin was removed: cells were washed once with 1 mL of calcium-free PBS, and 1 mL of Epilife media was added. Media from senescent keratinocytes were collected and immediately frozen at −80 °C, 72 h after induction of senescence with doxorubicin. Pierce BCA protein assay (Thermo Fisher, Cat# 23225) was conducted on cells for normalization purposes. To confirm senescence induction, senescence-associated β-galactosidase (SA-β-GAL), a biomarker of senescent cells, was stained using the Senescence Detection Kit from Abcam (Cat# ab65361) according to the manufacturer’s instructions.

### 4.6. Transcriptome Analysis of Normal and Doxorubicin-Induced Senescent Keratinocytes Using RNASeq

For transcriptomics experiments, HaCaT keratinocytes were seeded into 6-well tissue culture plate at 3 × 10^6^ cells per well and maintained in Epilife medium at 37 °C, 5% CO_2_, and 90% relative humidity. Three days after cells were plated, senescence was induced with 500 nM of doxorubicin hydrochloride for 24 h. Doxorubicin hydrochloride was removed: cells were washed once with 1 mL of calcium free PBS. RNA was harvested by adding 350 µL of RLT Buffer (Qiagen, Germantown, MD, USA) and lysates were transferred to tubes.

RNA sequencing was performed using Illumina Nextseq2000 platform (Illumina, San Diego, CA, USA). RNA (250 ng total RNA/sample) was polyA-selected, fragmented and converted to cDNA (Illumina Stranded mRNA Prep, P/N 20040534). cDNA fragments were adenylated, and anchors were ligated to the ends of the fragments to provide a binding site for unique dual index sequences (IDT^®^ for Illumina^®^ RNA UD Indexes, P/N 20040553). The resulting products were purified and amplified before being pooled for sequencing on the Illumina NextSeq2000 (NextSeq 2000 P3 Reagents, P/N 20040560). After sequencing, FASTQ files were trimmed by TrimGalore. Trimmed files were aligned to RefSeq GRCh38.p14 using STAR aligner via RSEM [45]. RSEM was then used to generate FPKM and count files. Deseq2 was used to calculate fold change and statistical significance between conditions (N = 4 for each group). Differentially expressed genes had Q-value <0.01, |FC| > 2, and FPKM > 1 in over half of the biological replicates in one condition being compared. Pathway analysis of differentially expressed genes was performed using QIAGEN’s Ingenuity Pathway Analysis (IPA). Select pathways predicted to be activated 24 h after dox treatment were visualized. IPA’s gene annotation was used to annotate genes as “extracellular space”-located and pathway design was used for network visualization. ToppFun functional enrichment analyses was used to determine which of the “extracellular space” annotated genes were also associated with cell growth (https://www.ebi.ac.uk/QuickGO/term/GO:0016049, accessed on 31 October 2025) and positive regulation of cell differentiation (https://www.ebi.ac.uk/QuickGO/term/GO:0045597, accessed on 31 October 2025) [46].

### 4.7. Melanocyte Dendricity Assay

Primary human melanocytes were plated into 96-well plates in complete Media 254CF (Thermo Fisher) and monitored with Incucyte Zoom (Sartorius, Göttingen, Germany) inside a 37 °C CO_2_ incubator. Dendricity was measured every 3 h (N = 24 for each group) after sample treatment using the dendrite length-to-cell body area metric from the IncuCyte ZOOM NeuroTrack software (version 1.4.0, Sartorius).

For statistical comparison, a mixed-effects model was fitted to the data, incorporating sample as a random effect and time and treatment as fixed effects, along with their interaction. To compare the effects of treatments at each time point, the R functions emmeans and pairs from the emmeans library were utilized. *p*-values were adjusted using the Bonferroni method to account for multiple comparisons, with the significance threshold set at *p* ≤ 0.05. Prior to modeling, the quality of the data was assessed by removing samples that were more than 1.5 times the interquartile range (IQR) away from the IQR of samples at the same time point and within the same treatment group.

### 4.8. IGFBP3 and NGF Protein Quantification in Culture Media via ELISA

Media from senescent keratinocytes and normal keratinocytes (N = 6 for each group) were analyzed for IGFBP3 level using the IGFBP3 R-plex kit from Meso Scale Discovery (Rockville, MD, USA) according to the manufacturer’s instructions. NGF levels were measured using the Human NGF ELISA kit from Sigma-Aldrich (Cat# RAB380). Total protein amount was measured using the Pierce BCA Kit (Thermo Fisher Cat# 23225) and was used to normalize the detected IGFBP3 or NGF amounts. For the statistical comparison, the normalized data were analyzed using Student’s *t*-test, with a significance threshold set at *p* ≤ 0.05.

### 4.9. Effect of IGFBP3 and NGF on Melanin Production and Melanocyte Dendricity

Human neonatal melanocytes were seeded into 6-well plates at a density of 500,000 cells per well in 2 mL of complete Melanocyte 254 medium, with 4 flasks per treatment group. Treatments were administered, and the melanocyte cultures were incubated for 72 h in a 37 °C CO_2_ incubator. Melanocytes were released with trypsin and concentrated into a pellet. 500 µL of cell lysis solution was added and plates were shaken for 10 min. A 100 µL lysate sample was plated in 96-well plate, and the optical density at 410 nm (OD410) was measured using a plate reader. Blanks containing cell lysis buffer were also measured, and these readings were subtracted from the treatment readings to provide Net OD410 values used to calculate melanin induction (N = 4 for each group). Melanocyte dendricity induction was performed as described in Section 4.7, with the addition of indicated amounts of IGFBP3 or NGF protein in the medium (after 48 h of treatment, N = 16 for each group). For both assays, statistical comparisons of treatment effect were performed using one-way ANOVA, followed by Tukey’s multiple comparisons test, with a significance threshold set at *p* ≤ 0.05.

### 4.10. Effect of Skin Care Compounds on the Release of IGFBP3 and NGF Proteins from Senescent Keratinocytes

The impact of skin care ingredients on the release of IGFBP3 and NGF proteins from senescent keratinocytes was assessed in vitro. IGFBP3 and NGF protein levels in the media from senescent keratinocytes were measured after 72 h of treatment with either niacinamide (pH 4 or 7), glabridin, sucrose dilaurate or tranexamic acid, using DMSO as a vehicle (including no treatment). Prior to treatment, the testing concentration for each ingredient was determined based on cell viability using the CyQuant^TM^ NF cell proliferation assay kit (Cat# C35006; Thermo Fisher), and the maximum non-cytotoxic concentration was used for the ligand protein release assay. Senescent keratinocytes were prepared by doxorubicin induction as described in Section 4.5, with the addition of the maximum non-cytotoxic concentration of the skin care ingredient (either a 1/50 dilution of 5% niacinamide at pH 7, a 1/50 dilution of 5% niacinamide at pH 4 adjusted with hydrochloric acid, 0.00125% glabridin, 0.001% sucrose dilaurate, or 0.1% tranexamic acid) when doxorubicin was added (N = 6 for each group). After 24 h, doxorubicin and skin care compounds were removed, the cells were washed, and fresh media along with fresh skin care compounds were added for additional 48 h (total 72 h of ingredient treatment). IGFBP3 and NGF proteins released in the media were analyzed by using ELISA kits and normalized to the total protein amount as described in Section 4.8. The detected amounts were normalized to the senescent keratinocyte no-treatment control levels. For statistical comparison, the data were analyzed using one-way ANOVA, followed by Tukey’s multiple comparisons test, with a significance threshold set at *p* ≤ 0.05.

## Data Availability

The raw data supporting the conclusions of this article will be made available by the authors on request.

## Supplementary Materials

The following supporting information can be downloaded at: https://www.mdpi.com/article/10.3390/ijms262110724/s1.

## Abbreviations

The following abbreviations are used in this manuscript:

SASP	Senescence-associated secretory phenotype
IGFBP3	Insulin-like growth factor-binding protein-3
NGF	Nerve growth factor
UV	Ultraviolet
SD	Standard deviation
RT	Room temperature
