# Supplementary material for: Over-Represented Senescent Keratinocytes in Hyperpigmented Spots Promote Melanocyte Activation via IGFBP3 and NGF
Source: Int J Mol Sci. 2025 Nov 4;26(21):10724. doi: 10.3390/ijms262110724 (PMC12608426; doi:10.3390/ijms262110724)
Supplement: Supplementary file 1 [file ijms-26-10724-s001.zip › ijms-3904597-supplementary.pdf]

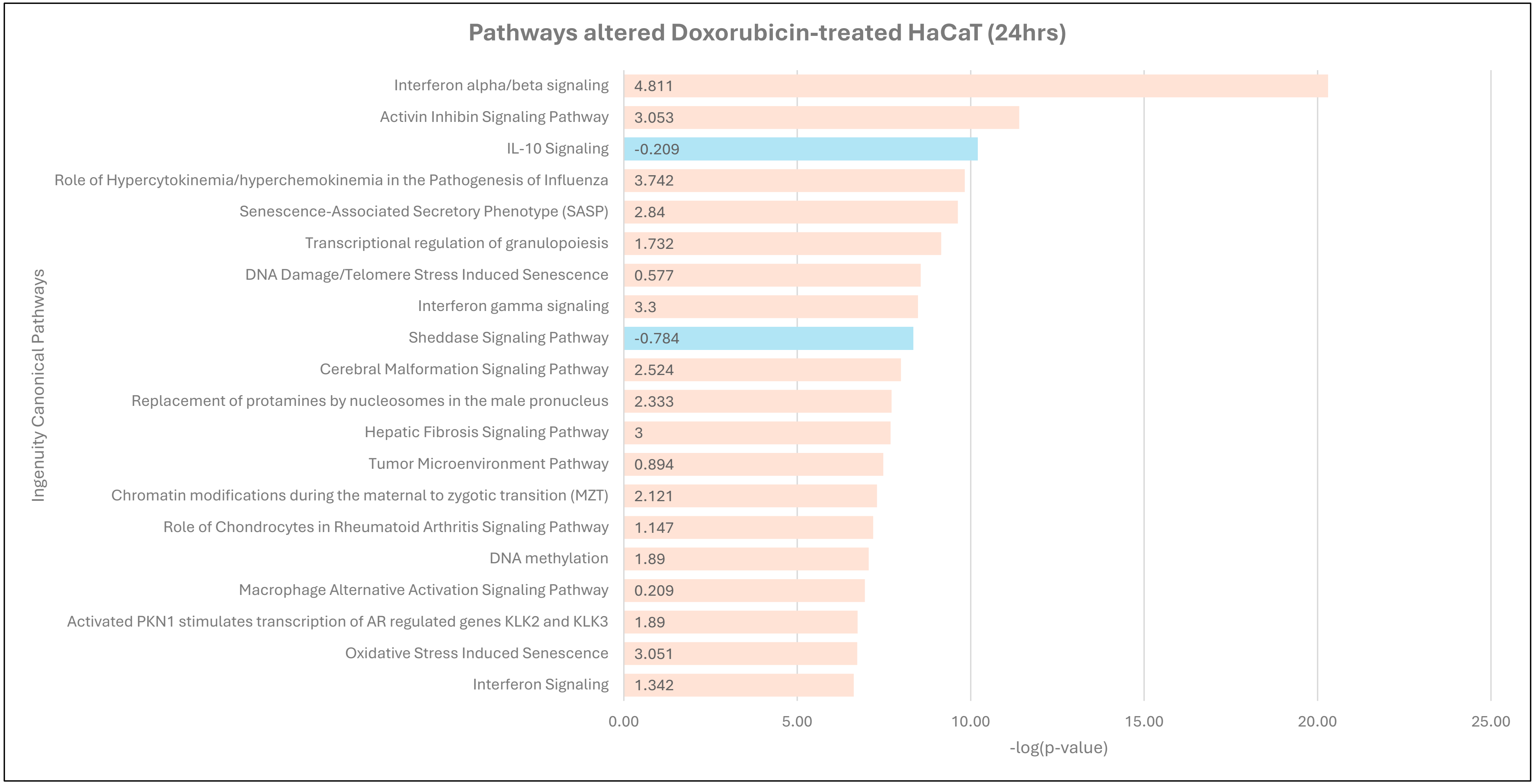

Figure S1. RNAseq analysis was performed 24 hours after 500 nM doxorubicin treatment, comparing treated and untreated HaCaT keratinocytes. Genes differentially regulated along with fold change were used as input for IPA's Pathway Analyses. Top 20 significantly altered pathways from IPA are displayed. IPA predicted activation of Interferon signaling and Senescence related pathways. Activation score displayed within corresponding bar. RNAseq n=4. Differentially regulated genes, Q-value <0.01, |FC|>2, and FPKM>1 in over half of the biological replicates in one condition.
